# Decision Confidence Assessment in Multi-Class Classification

**DOI:** 10.3390/s21113834

**Published:** 2021-06-01

**Authors:** Michał Bukowski, Jarosław Kurek, Izabella Antoniuk, Albina Jegorowa

**Affiliations:** 1 Institute of Information Technology, Warsaw University of Life Sciences, Nowoursynowska 159, 02-776 Warsaw, Poland; michal.bukowski@buksoft.pl (M.B.); izabella_antoniuk@sggw.edu.pl (I.A.); 2 Institute of Wood Sciences and Furniture, Warsaw University of Life Sciences, Nowoursynowska 159, 02-776 Warsaw, Poland; albina_jegorowa@sggw.edu.pl

**Keywords:** confidence classification, confidence functions, multi-class classification, tool condition monitoring, laminated chipboard

## Abstract

This paper presents a novel approach to the assessment of decision confidence when multi-class recognition is concerned. When many classification problems are considered, while eliminating human interaction with the system might be one goal, it is not the only possible option—lessening the workload of human experts can also bring huge improvement to the production process. The presented approach focuses on providing a tool that will significantly decrease the amount of work that the human expert needs to conduct while evaluating different samples. Instead of hard classification, which assigns a single label to each class, the described solution focuses on evaluating each case in terms of decision confidence—checking how sure the classifier is in the case of the currently processed example, and deciding if the final classification should be performed, or if the sample should instead be manually evaluated by a human expert. The method can be easily adjusted to any number of classes. It can also focus either on the classification accuracy or coverage of the used dataset, depending on user preferences. Different confidence functions are evaluated in that aspect. The results obtained during experiments meet the initial criteria, providing an acceptable quality for the final solution.

## 1. Introduction

Ensuring an efficient and smooth flow of production processes can be challenging, time-consuming, and, at times, also problematic. For example, in the wood industry, from the many tasks that need to be monitored, some of them will require specialized knowledge and precision, while others will use up a significant amount of time, and there are quite a lot of activities that combine all of those features. One of such tasks concerns evaluating the state of drills in the manufacturing process, which is a subset of problems widely known as tool condition monitoring. Usually, when manually performed, this task requires stopping the production process in order to evaluate individual tools. At the same time, a human expert is required to check used elements, without any indication to its actual state. Due to that, unnecessary downtime may occur, when it could have been avoided if the entire process had been, at least partially, automated.

When it comes to tool evaluation, many different approaches have been considered to either speed up the process, or avoid human intervention in general. For example, the main focus may include evaluating the state of elements without interrupting the actual manufacturing process, as presented in [1]. Most basic and commonly used approaches, such as the one presented in [2], measure different signals, such as vibration, noise, acoustic emission, cutting torque, feed force, and others, in order to evaluate the tool state. Similar approaches were used in [3], where data were extracted both from signal and frequency domains, along with wavelet coefficients, all in order to evaluate the obtained elements automatically, checking how relevant each item was to the selected problem. Further, in [4], the authors used different signals, over a wide range of cutting conditions, using a back-propagation neural network to predict the flank wear of a drill bit. Another approach which relies heavily on different sensors is presented in [5]. In this case, various sensors were used to collect data, which were later integrated using a fuzzy logic model.

While sensor-based approaches are quite widely used, they are not always the best solutions. First of all, the equipment required to even start taking different measurements tends to be quite expensive and would also require lengthy calibration, which, if conducted incorrectly, can affect the resulting accuracy. Furthermore, a setup which contains multiple sensors might be difficult to integrate into an actual work environment without affecting the production process. With all those problems, as well as some additional requirements that might appear in different industries, regarding the desired accuracy or some additional properties of the final solution, such as limiting the number of critical errors (which corresponds to any mistakes between border classes of tool wear), a simpler input might be required.

In some previous works, images were used as a base for drill state evaluation, using various machine learning algorithms. Such solution was considered in [6,7]. In those cases, the specialized sensors were dropped entirely, and instead of signals, images of drilled holes were used for evaluation purposes, requiring only a simple camera to obtain them. The presented solutions are based mainly on convolutional neural networks (CNNs, which have the additional advantage of not requiring any specialized, diagnostic features; those networks are also considered top solutions in the case of image recognition, as mentioned in [8]). The first of the two approaches uses the data augmentation technique to achieve dataset expansion without the need for additional samples, combined with a transferred learning methodology. Accuracy of 93% was achieved here, without the need for a complicated sensor setup. In the case of the second solution, a classifier ensemble was used to further increase the overall classification rate, exceeding a 95% accuracy rate. There are also some recent approaches that incorporate similar methodologies. In [9], various CNN networks were tested and evaluated to prepare an improved approach that focused more on limiting critical errors that the classifier makes. In another solution, presented in [10], a Siamese network was applied to the same problem, which is a new, CNN-based methodology. In both approaches, the window parameter, which included consecutive images of holes drilled in sequence, was used to further increase the achieved results. Finally, in [11], a more time-efficient approach was presented, this time using image color distribution, with an assumption that, after converting the image to gray-scale, there will be more pixels with mid-range values within images representing holes drilled by more used tools. All those solutions achieve high accuracy results and a relatively low amount of critical errors.

What can be noted is that while most of the presented solutions take into account the manufacturer requirements, they also have some drawbacks. First of all, in the case of more difficult examples, the solutions tend to make different errors in the final classification (some more severe than others). Second of all, the manufacturer cannot easily switch between different metrics used to evaluate the final solution. Those drawbacks led to the current approach, in which images are still used as input data, but instead of using a hard classification model, which assigns classes to each presented example, a more elastic approach is incorporated. Instead of classifying each sample as belonging to one of the classes (green for a tool which is new, red for a tool that should be discarded, and yellow for one that requires further evaluation), a confidence metric is incorporated to inform the user how exact the current classification is. Samples can then either be further classified or discarded and assigned to the human expert for that purpose. Furthermore, the solution can be adjusted to focus either on accuracy or the dataset coverage—since different industries might have varying requirements in regard to those aspects, such approach provides the user with more control over how the presented solution works. It will also allow for easier adaptation to chosen problems.

The novelty of this work is that it provides a robust way to quantify the uncertainty of any multi-class classification into a confidence parameter that allows us to discriminate some observations with low confidence in order to increase performance metrics for the rest of the observations. This approach allows easily combining human expert knowledge and algorithm ways of classification and can be added on top of the multi-class classifier.

## 2. Methodology

In previous work (see [11]), it was noted that while using a set of images converted to gray-scale, the amount of pixels placed in the mid ranges can be used to evaluate the state of the drill that was used to prepare the hole shown on each image. The research presented in this article continues on this assumption, but instead of hard classification, where each image is assigned a single class, the samples are evaluated in terms of decision confidence.

The presented confidence evaluation process consists of a few steps, involving initial data processing, model preparation, and the confidence function itself.

### 2.1. Dataset

The dataset used in the current experiments contained a total of 8526 images showing holes drilled by steadily declining tools. For the initial class evaluations, the resulting sample set was manually labeled. In the case of the presented dataset, external corner wear—W (mm) was used as a decisive factor for assigning a class to each image. This parameter was measured using a workshop microscope, TM-505 Multitoyo, Kawasaki, Japan. According to experts in this field, the parameter ranges were established as follows:

- W < 0.2 mm—drill classified as green;
- 0.2 mm < W <  0.35 mm—drill classified as yellow;
- 0.35 mm <  W—drill classified as red.

From those images, 3780 were classified as the green class, 2800 samples were classified as the yellow class, and 1946 represented the red class. Images were chosen as input data, since, while showing the declining state of the drill (edges tend to be more jagged in the case of more used tools than in the case of new ones), they do not require a significant amount of time to obtain, and the acquisition process itself can be adjusted to the specific needs of each manufacturer. All samples used in the current research were obtained in cooperation with the Institute of Wood Sciences and Furniture at Warsaw University of Life Sciences. The summary of the dataset is presented in Table 1 below.

**Figure 1:**
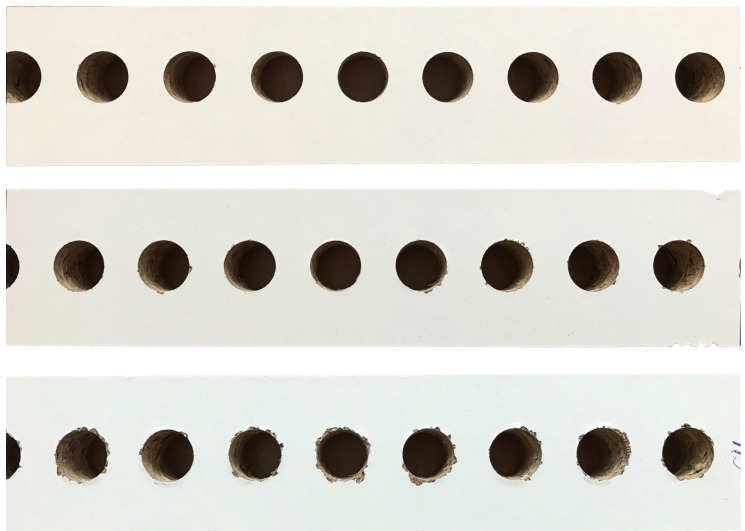
Images showing photographed samples after division into smaller elements, representing holes made by drills with different drill wear classifications: green (**top**), yellow (**middle**), and red (**bottom**).

**Figure 2:**
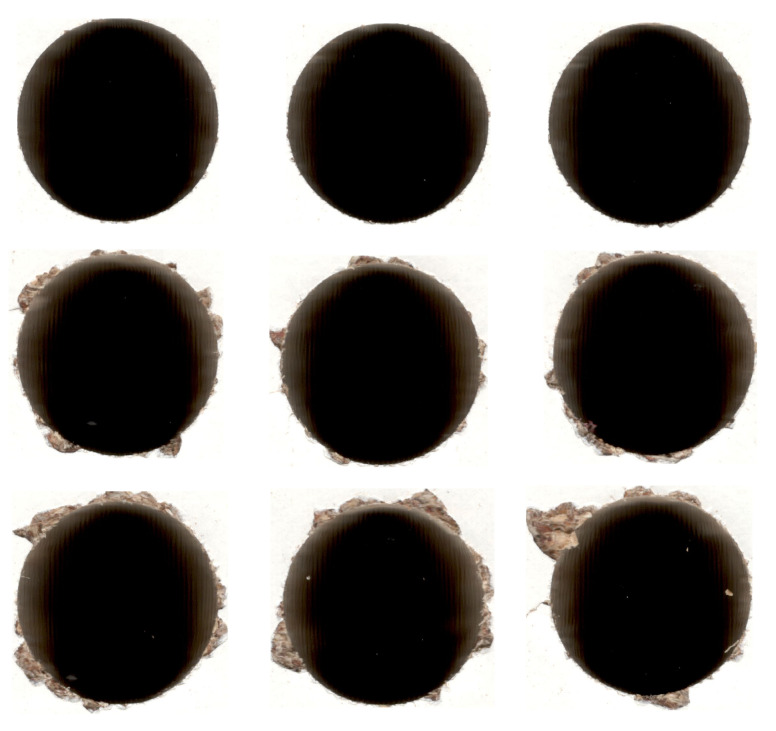
Images obtained after initial processing which are used as an input to the main procedure: green (**top**), yellow (**middle**), and red (**bottom**).

Holes were drilled with a standard CNC vertical machining center, Busellato Jet 100 (Busellato, Thiene, Italy). To ensure that the entire setup is as close to the potential manufacturer requirements as possible, the drilled material was a material typically used in the furniture industry, laminated chipboard U511SM Swiss Krono Group (Swiss Krono Sp. z o.o., Żary, Poland). The drill used for this application was a 12 mm Faba WP01 (Faba SA, Baboszewo, Poland) with a tungsten carbide tip. Initial test piece dimensions were 2500 mm × 300 mm × 18 mm. To acquire the actual images, they were later divided into smaller ones, which were separately photographed. Example fragments representing each of the recognized classes are presented in Figure 1. Final images, representing one hole each, were obtained using a custom script, which extracted the desired area and saved it in separate images with three RGB color channels. The images were stored in the exact order in which they were made, facilitating easier evaluation of the obtained results, but the time series structure of the obtained data was not incorporated into the current solution. Example images showing the input images used by the presented procedure are shown in Figure 2.

All the images in the current dataset were manually labeled as one of recognized classes by a human expert and later used by the prepared model.

### 2.2. Model

Images converted to gray-scale using ITU-R 601-2 luma transform specification were the input data for the following algorithm steps. During model preparation, the initial classification based on the overall grouping of pixels was conducted. The initial research presented in [11] showed that, although the images with holes of degrading quality show a steady increase in gray pixels (the pixels in each image were divided into three groups for that process—black for the hole, white for the laminated chipboard surface, and gray for the hole edge), there is no clear border between each class; hence, the images cannot be easily classified using only that count. At the same time, the general relation between the number of gray pixels and the quality of the drilled hole still remained, with images of holes belonging to the green class having significantly less gray pixels than those that belonged to the yellow or red classes.

During this initial step, the image preparation was conducted. Original images were represented in RGB and varied in size (the custom script prepared for this phase focused on cutting out the fragment of the image containing the hole, with the edges, and with as little detail from the surrounding sample as possible, but including any jagged parts of the hole edge). Due to that variation, in the first step, images were resized to 256 × 256 pixels to make sure that they have a uniform size. Additionally, since the information regarding the state of the hole edge does not require color values, the images were converted to gray-scale.

The next step involved counting occurrences of each pixel value and normalizing them to fit in the 0–1 range. This was accomplished by a simple operation of dividing each pixel count by the total number of pixels in the image. From those counts, an array containing 256 pixel values was prepared, which was later used as an input to the next part of the model. This element used Light Gradient Boosting Machine (also LGBM or Light GBM, described in [12]). LGBM uses tree-based learning, which grows trees vertically, with the maximal delta loss for leaves, and can handle larger datasets while using less memory. This approach also focuses on accuracy and has an efficiency parameter used as one of the main quality indicators. During experiments, 15 rounds of Bayesian optimization were used to choose and optimize hyperparameters with a multi-log loss metric. Data obtained from this element were later used in the following steps of the presented procedure.

In order to obtain probabilities with a window size of 1 (meaning that only a single image is taken into account, and not a sequence of images), the 5-fold cross-validation method was used. The presented method can also be easily expanded to include larger windows. Meanwhile, the baseline accuracy for the chosen set of parameters was 0.67 (these results were obtained in previous work [11], where no feature selection approach with a window equal to 1 achieved exactly the same result). Given the probabilities’ distribution, achieved by 5-fold cross-validation, we calculate the confidences for each of the 4 different confidence functions defined in Section 3. For each of those 4 results, we calculate different metrics of how well they achieved their goal of measuring confidence, which are also defined in Section 3. We compare the results of those metrics in Section 4.

### 2.3. Problem Formulation

The main metrics considered with the current classification model were accuracy and the number of severe (red–green) errors. In order to increase both of them, several approaches can be adapted, including using a set of subsequent images instead of single ones, as conducted in previous authors’ work [9] using window parameters. After that, the dominant value of all classifications can be used. This method, although it increases the classification rate in general, is only suitable for some problems, especially since it complicates the deployment of the classifier iinto the manufacturing process (the industry needs to be able to produce subsequent images during production).

In the current approach, instead of hard classification, where an image is assigned to one of the recognized classes, an additional class or state was added, making it possible to return an “I don’t know” or an “undefined” pseudo-class, where some observations will not be classified at all. Later on, those samples labeled as “undefined” can be further examined by human experts. While this approach is not fully automatic, it can eliminate the majority of observations with a clear classification, leaving only harder and more interesting examples for manual evaluation, possibly resulting in better performance of the entire solution. The folds are the same as in Table 1; therefore, this process is based on 5-fold cross-validation. The overall structure of the presented solution is shown in Listing 1, where classification and calculating confidence are presented, and in Listing 2, where different confidence metrics are calculated.

**Listing 1:**
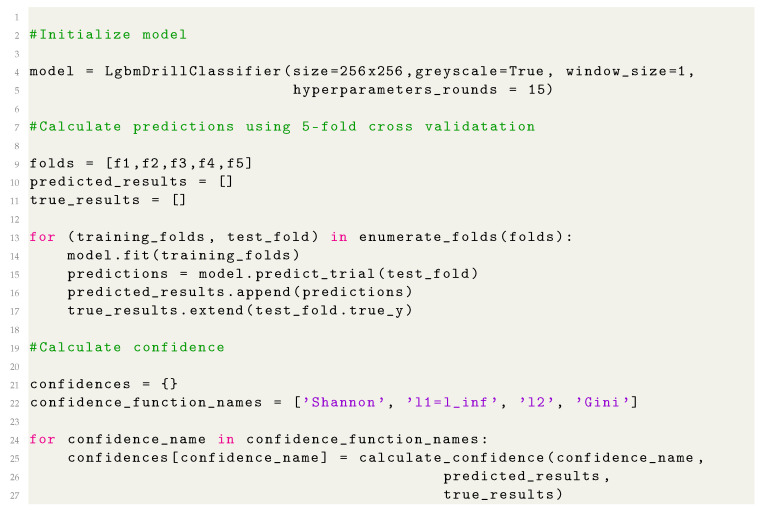
Classifying observations, and calculating confidence.

**Listing 2:**
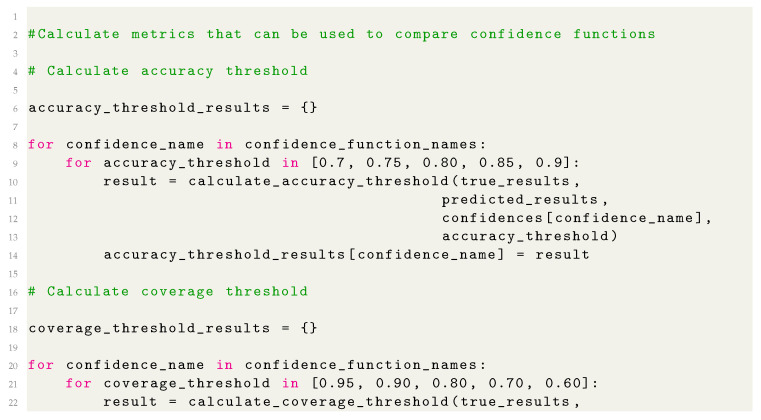

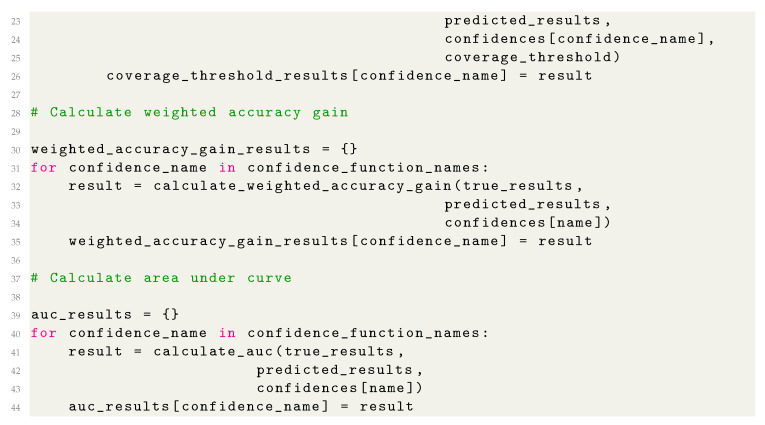
Calculating confidence metrics.

## 3. Confidence Function

The approach presented in this paper is based on a confidence function, which will describe how sure used the model is of the presented classification. The results which have low confidence can be discarded, hence leaving only those with higher values in that aspect. By using such filtered samples, it is believed that better results can be obtained when compared with using the entire dataset with an unsure classification. An ideal situation will, for example, drop 2% of the least confident results, boosting the actual accuracy by around 10%.

### 3.1. Confidence Function Constraints

In order to use some methods as confidence functions, a set of constraints needs to be defined first. Assume that a result’s probability vector of a multi-classification problem with a number of classes of *n* ≥ 3 is given with probabilities obtained using the softmax function (which is a common practice in neural networks and other models [13]).

To achieve comparable results for different confidence functions, the function should be able to transform an *n* element vector of probabilities into a single value in the range [0.0, 1.0].

The confidence function Conf should satisfy three constraints:

(1) $$Conf(v_{eq})=0$$

(2) $$Conf(v_{unit})=1$$

(3) $$0<Conf\left(v\right)<1\;\forall v\in V_{n}\backslash \left\{v_{eq},v_{unit}\right\}$$

where $v_{eq}$ is the probability vector containing equal probabilities, $v_{unit}$ is the probability vector with all but one element equal to 0, and $V_{n}$ is the set of all probability vectors with length *n*.

Given some confidence threshold *t*, all observations that have a confidence score lower than *t* are categorized with the “undefined” pseudo-class. The coverage *c* is the fraction of all observations for some threshold *t* that are still normally classified, and we will denote the accuracy of that classification as *a*. Increasing the threshold should increase accuracy, but it will decrease coverage.

### 3.2. Confidence Function Candidates

While the constraints and some general assumptions for the presented model were defined, it still requires some candidates for the confidence function to be pointed out. For the initial approach, a total of three methods were considered: Shannon, Gini, and Norm confidence, which are outlined below.

#### 3.2.1. Shannon-Based Confidence

Shannon entropy is a good candidate for confidence, as it is used as an inequality measure [14]. It can be defined as presented in Equation (4).

(4) $$Conf_{Shannon}\left(v_{n}\right)=1.0-H_{n}\left(v_{n}\right)$$

where $v_{n}$ is an *n* dimensional probability vector, and $H_{n}$ is the entropy with a logarithm base equal to *n*. It satisfies all constraints because the maximal possible entropy achieved with $v_{eq}$ is 1, and for $H_{n}\left(v_{unit}\right)$, it is 0.

#### 3.2.2. Gini-Based Confidence

Another measure of inequality [15] is the Gini coefficient. In order to satisfy the chosen constraints, they should be normalized by the Gini coefficient of the unitary vector $v_{unit}$. Gini confidence can then be defined as shown in Equation (5).

(5) $$Conf_{Gini}\left(v_{n}\right)=Gini\left(v_{n}\right)/Gini\left(v_{unit}\right)$$

As $Gini(v_{eq})$ is 0, and maximal inequality is achieved by $v_{unit}$, this confidence measure also satisfies all constraints.

#### 3.2.3. Norm-Based Confidence

To use a slightly different approach, the inequality of a given prediction can also be measured as the distance to the closest unitary vector. In order to satisfy the constraints, the distance needs to be adjusted, considering the maximal possible distance, which is the distance from $v_{unit}$ to $v_{eq}$. To choose the distance metric, standard $l_{1}$, $l_{2}$, or $l_{inf}$ norms can be used, as shown in Equation (6).

(6) $$Conf_{norm}\left(v_{n}\right)={max}_{0\leq i\leq n}\left(1-\frac{norm(v_{n}-{\left[0,\cdots 1_{i},\cdots ,0\right]}_{n})}{norm(v_{unit}-v_{eq})}\right)$$

The maximum norm $l_{inf}$ is the baseline comparison of all confidence functions (see Equation (7)). It is the simplest one of the presented functions, and it just measures the distance between the maximum probability and 1.0, standardizing it to be between [0.0, 1.0] so it can fulfill the constraint set.

It is worth noting that with that scaling, $l_{inf}$-based confidence gives the same results as $l_{1}$-based confidence; therefore, a baseline value can be obtained using either of those functions.

(7) $$Conf_{l_{1}}=Conf_{l_{inf}}$$

### 3.3. Comparing Confidence Functions

To select the best confidence function, which will be one that maximizes the accuracy *a* and coverage *c* for all thresholds *t*, a direct comparison of the chosen confidence functions is required for given model outputs. One additional factor that also needs to be included is the actual gain the confidence function gives for the current approach. This is achieved by comparing the confidence threshold accuracy with the default approach which does not use any confidence at all (default accuracy for the presented model).

#### 3.3.1. Accuracy Threshold

Since the presented approach aims at being as versatile as possible, it is worth noting that depending on the specific application, the accuracy constraints might differ, requiring the solution to achieve specific values in that aspect. For that approach, the best confidence function would be one that, for the chosen accuracy threshold *a*, will achieve a confidence threshold *t* that it ensures the best coverage *c*.

In the presented case, the default accuracy of the used classifier is 0.67, and the goal accuracy is 0.80. The threshold *t* for Shannon-based confidence is 0.33, which corresponds to 0.80 accuracy *a* and 0.55 coverage *c*. For $l_{2}$-based confidence, the threshold *t* will be 0.41 with 0.46 coverage *c*. Therefore, in that problem, Shannon-based confidence is a better confidence function than $l_{2}$-based confidence.

#### 3.3.2. Coverage Threshold

Another approach to this problem would consider, instead, that the confidence function should maximize the accuracy *a* for observations above the confidence threshold *t* corresponding to a given coverage threshold *c*. In general, this would correspond to eliminating the most problematic (1−c) fraction of cases from the set, and maximizing the accuracy on the rest of the observations.

For example, in the presented model, let us assume a 0.9 coverage threshold is our goal. With Gini-based confidence, the corresponding confidence threshold *t* would be 0.42 with accuracy *a* equal to 0.69. With the same requirements, for $l_{1}$-based confidence, the confidence threshold *t* would be 0.02 with accuracy *a* of 0.67.

#### 3.3.3. Weighted Accuracy Gain

Weighted accuracy gain measures the weighted sum of the difference between different threshold accuracies and default classifier accuracies, as shown in Equation (8).

(8) $$Wag=\frac{\sum_{t=0.0}^{1.0}\left(a_{t}-a_{0}\right)\times c_{t}}{n}$$

where $a_{t}$ is accuracy with a confidence threshold *t*, $a_{0}$ is accuracy with a confidence threshold of 0, which corresponds to the baseline classifier accuracy, $c_{t}$ is coverage with a confidence threshold *t*, and *n* is the number of thresholds considered.

#### 3.3.4. Confidence Area under Curve

In the case when no accuracy threshold is given, several confidence thresholds can be checked instead, and the function maximizing both accuracy and coverage should be chosen. This problem is similar in formulation to the receiver operating characteristic area under the curve—roc_auc [16]—and we will be using the auc shortcut in the next sections.

**Figure 3:**
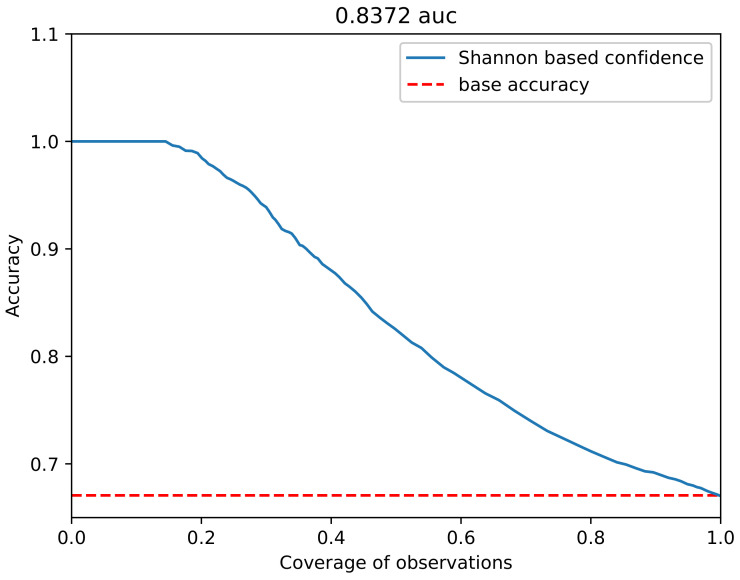
Plot presenting confidence curve for Shannon-based confidence, where it achieves a 0.8372 auc score.

The area under the confidence curve (as shown in Figure 3) can be calculated using the trapezoid rule. The used points were constructed in pairs (*a*, *c*) that correspond, respectively, to accuracy and coverage for each of the confidence thresholds *t*, from 0 to 1.0. It was assumed that an accuracy with confidence of 1.0 is also one of the thresholds with the lowest coverage. The baseline for this is also the accuracy result of the initial model, which does not include any type of confidence.

## 4. Results and Discussion

The process of calculating results for different confidence functions is shown in Listing 2 and is based on the dataset presented in Table 1 using the 5-fold cross-validation presented in Listing 1.

**Figure 4:**
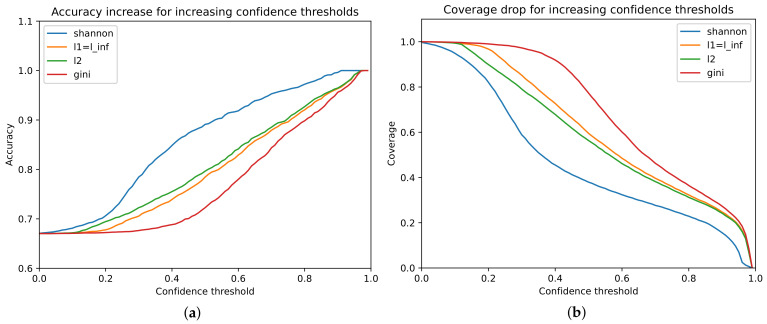
Accuracy (**a**) and coverage (**b**) curves for evaluated functions.

For each confidence function, different accuracy and confidence threshold functions were used. There is an expected trade-off between accuracy gain and coverage drop. For example, the Shannon-based confidence accuracy change is presented in Figure 4a, represented by the blue line. As it can be seen, it comes with a drop in the coverage, as the same colored line representing this function in Figure 4b is the lowest one. All norm-based confidences behave similarly in that aspect. In the case of the general accuracy–coverage, Shannon-based confidence and Gini-based confidence seem to represent opposite trade-off values; therefore, their usage should be adjusted accordingly.

### 4.1. Comparison of Accuracy Threshold

The main goal of the presented research is to increase the accuracy and keep high coverage *c*. The first method of comparison focuses on the coverage of observations that is kept given some accuracy threshold. The best results for different accuracy thresholds *a* were obtained by different functions, with Gini-based confidence being the only exception to that (see Table 2). The used baseline $l_{inf}$ was the best in two of five cases. While high accuracy can be obtained, the methods that actually achieve it also need to sacrifice a significant portion of coverage *c*. The highest value of coverage for the 90% threshold was achieved for Shannon-based confidence but covered only 36% of the used dataset. In comparison, the accuracy rate of 70% was able to cover over 87% of the data for the $l_{1}$ = $l_{inf}$ function. It is also worth noting that different functions performed best for different accuracy thresholds; therefore, some additional improvement can be achieved by using the best performing function for the current setup.

### 4.2. Comparison of Coverage Threshold

The problem presented in this paper can also be approached from the coverage threshold point of view. If the goal of classification, instead of achieving a specific accuracy, is more focused on including as many samples as possible (with only a specified fraction of examples being evaluated by a human expert), the coverage threshold approach should be used. The achieved results for different coverage parameters are presented in Table 3. In this case, the baseline $l_{inf}$ metric is the best two out of five times. The best results are obtained for the $l_{2}$ norm, which wins three out of five times; however, differences for each coverage threshold *c* are often negligible.

### 4.3. Comparison of Weighted Accuracy Gain

For this metric, the baseline $l_{inf}$ performs significantly worse than the other metrics. The best result is achieved by Shannon-based confidence, and second place is taken by $l_{2}$. Gini-based confidence again is not the best solution. Results for the weighted accuracy gain comparison are presented in Table 4.

### 4.4. Comparison of Area under Confidence Curve

**Figure 5:**
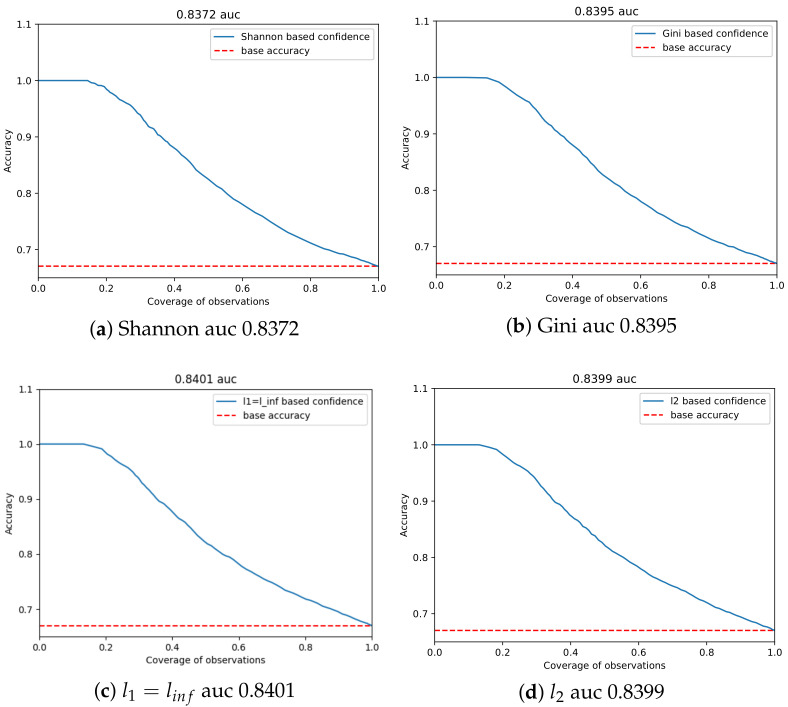
Confidence threshold curves comparison.

In the case of the area under the confidence curve comparison, the obtained results are very indecisive, presenting similar qualities for all used functions. As it can be seen in Figure 5, all curves look very similar, and differences between all norms are negligible. The baseline confidence norm $l_{inf}$ has the highest auc result of 0.401 (see Table 5), but differences between the functions are not significant. Further investigation is needed to check if this is the case for all models.

### 4.5. Error Rate Evaluation

When it comes to the relation between accuracy and the used coverage threshold, one additional parameter needs to be evaluated. When it comes to overall requirements in different industries, usually mistakes between border classes are the most costly ones (green and red in the case of drill wear classification, which was presented in this paper). Such errors should be avoided in general, and the coverage parameter presented in this paper was introduced in order to discard as many unsure examples (for later classification by a human expert) as possible, in order to increase the classification accuracy and reduce the number of severe errors. In order to evaluate the impact of coverage on the number of critical errors that the classifier makes, the Shannon-based confidence from Figure 3 will be used.

**Figure 6:**
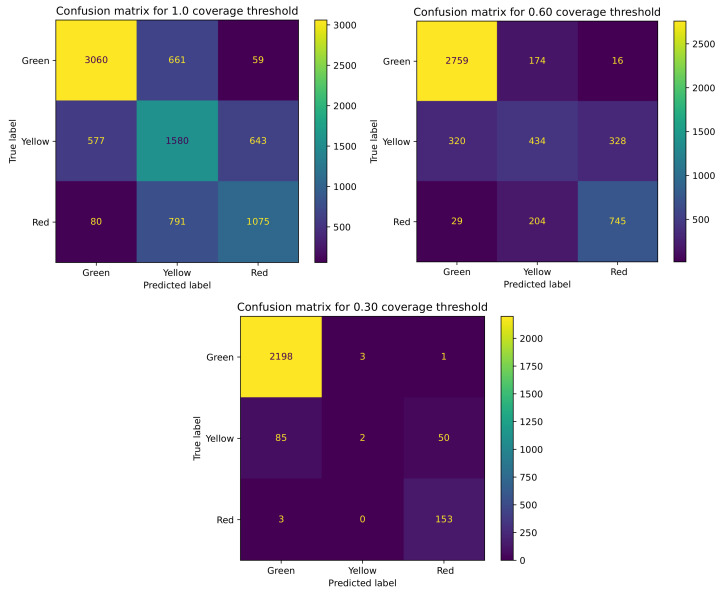
Confusion matrix obtained for different coverage thresholds: full dataset coverage and a baseline value for the critical error rate (**top left**), 60% coverage (**top right**), and 30% coverage (**bottom**).

Figure 6 shows confusion matrixes for three coverage thresholds, starting with the classification representing the full dataset, and finishing with coverage of 30%. As it can be seen, while the coverage decreases, the number of critical errors (red–green or green–red) between border classes diminishes at the same time. For the initial, baseline setup, with full dataset coverage (which can also be denoted as an approach that does not use confidence at all), the overall number of critical errors equaled 159, with 80 cases of the red class classified as green and 59 green samples classified as red. This number decreases to a total of 45 critical errors when the dataset coverage decreases to 60% (29 red–green and 16 green–red misclassifications). The Shannon-based confidence function mostly removes observations with the yellow class. With this coverage threshold, we keep 50% of red observations, 39% of yellow observations, and 78% of green observations. The number of critical errors is even lower for the 30% coverage threshold, with only four critical errors (three red–green and one green–red). This threshold removes the yellow observations almost completely.

### 4.6. Discussion

The problem which confidence calculation is trying to solve can be commonly seen in our world. The machine learning aid in diagnosing cancer can be a great aid if the expected accuracy of the used model can be controlled. The coverage threshold can be adjusted for human resources in different companies, thus allowing easy scaling of human–computer hybrid systems. It also aligns with the trend of explainable artificial intelligence [17], where the confidence of each prediction is a very good parameter outlining the model transparency.

For the four different confidence functions and the four different metrics, there is no clear winner. The used baseline $l_{inf}$-based confidence performed well in three out of four metrics and thus is a good, robust confidence function. However, if the focus is put on a particular metric, Shannon-based confidence and $l_{2}$-based confidence can be better solutions, depending on the chosen parameters. The Gini coefficient, which is often used as an inequality metric, performed poor as a confidence function, which is a surprising result.

There are other approaches that are similar to the confidence-based approach presented in this work. Bayesian networks, as presented in [18], can also be used to discriminate some observations into the “undefined” class. The Bayesian approach requires changing the underlying parameters into a parameter distribution, and sampling results from a posterior distribution. This, however, requires deep modification in the underlying model and is considerably slower due to the need for sampling from several different distributions.

Another approach that can also incorporate the “undefined” class is fuzzy classification [19]. Observations with low membership values with each class can be labeled as “undefined”. There is also some work that defines confidence in fuzzy classification terms [20]. The caveat in this approach is that it requires entirely different problem formulations and datasets and thus cannot be easily combined with prior models.

## 5. Conclusions

In this article, a novel and adaptable classification algorithm was described. While the presented solution was mainly applied to drill wear classification, it is not limited to this task. Instead of focusing on hard classification, transferring to assigning a single class to each example, the method focuses more on evaluating confidence for each considered sample. While there is no clear winner in the evaluated confidence function set, the performance of at least one function for each accuracy or coverage threshold was at least acceptable. For some cases, the differences between different functions were negligible, while their performance was still satisfactory.

Even in its current state, the presented solution is quite versatile and can be easily adapted to any number of recognized classes. Furthermore, due to the confidence metrics applied, the model can be better evaluated in that aspect, pointing out how sure the classifier is when assigning a certain class to each sample. As an additional feature, depending on the manufacturer requirements, the method can focus more on obtaining the required accuracy rate, or covering a chosen fraction of samples. Finally, by discarding some of the examples, labeling them as too complicated for automatic classification (for the cases when the metric will show a confidence below the assigned threshold), and evaluating them by manual experts, the accuracy rate of the entire solution should perform better, avoiding severe errors which tend to be a problem with fully automatic solutions. All of the above features expand the available applications of the prepared algorithm. Additionally, when combined with a possible focus on either accuracy or dataset coverage, the overall functionality makes the presented solution more suited for various classification tasks that may appear in the wood and other industries.

## Figures and Tables

**Table 1:** Dataset structure.

Class	Fold 1	Fold 2	Fold 3	Fold 4	Fold 5	Total
Green	840	840	700	840	560	3780
Yellow	420	700	560	560	560	2800
Red	406	280	420	280	560	1946
Total	1666	1820	1680	1680	1680	8526

**Table 2:** Comparison of dataset coverage for different accuracy thresholds $a_{t}$ for each evaluated confidence function.

Confidence Function	$a_{t}$ = 0.70	$a_{t}$ = 0.75	$a_{t}$ = 0.80	$a_{t}$ = 0.85	$a_{t}$ = 0.90
Shannon	0.8403	0.6586	0.5389	0.4464	0.3611
$l_{1}$ = $l_{inf}$	0.8770	0.6929	0.5463	0.4429	0.3500
$l_{2}$	0.8692	0.6832	0.5493	0.4504	0.3456
Gini	0.8586	0.6661	0.5385	0.4424	0.3554

**Table 3:** Comparison of accuracy obtained by the evaluated confidence functions for different coverage thresholds $c_{t}$.

Confidence Function	$c_{t}$ = 0.95	$c_{t}$ = 0.90	$c_{t}$ = 0.80	$c_{t}$ = 0.70	$c_{t}$ = 0.60
Shannon	0.6799	0.6895	0.7063	0.7400	0.7756
$l_{1}$ = $l_{inf}$	0.6820	0.6945	0.7175	0.7457	0.7815
$l_{2}$	0.6807	0.6940	0.7182	0.7486	0.7823
Gini	0.6810	0.6895	0.7123	0.7426	0.7799

**Table 4:** Comparison of weighted accuracy gain with different confidence functions.

Confidence Function	Weighted Accuracy Gain
Shannon	0.0550
$l_{1}$ = $l_{inf}$	0.0491
$l_{2}$	0.0525
Gini	0.0387

**Table 5:** Comparison of threshold area under the curve with different confidence functions.

Confidence Function	Area under Curve
Shannon	0.8372
$l_{1}$ = $l_{inf}$	0.8401
$l_{2}$	0.8399
Gini	0.8395

## Data Availability

Data available on request due to privacy restrictions.
